# Cultural adaptation and psychometric evaluation of the Kinyarwanda version of the diabetes-39 (D-39) questionnaire

**DOI:** 10.1186/s12955-022-02034-5

**Published:** 2022-08-16

**Authors:** Jean Paul Uwizihiwe, Charilaos Lygidakis, Michela Bia, Damas Dukundane, Brenda Asiimwe-Kateera, Sabin Nsanzimana, Claus Vögele, Per Kallestrup

**Affiliations:** 1 Centre for Global Health, Department of Public Health, Aarhus University, Bartholins Allé 2, 8000 Aarhus C, Denmark; 2 College of Medicine and Health Sciences, University of Rwanda, Kigali, Rwanda; 3 Department of Behavioural and Cognitive Sciences, University of Luxembourg, Esch-sur-Alzette, Luxembourg; 4 Luxembourg Institute of Socio-Economic Research (LISER), Esch-sur-Alzette, Luxembourg; 5 Butaro Cancer Centre of Excellence, Burera, Rwanda; 6 AIDS Healthcare Foundation (AHF), Kigali, Rwanda; 7 Rwanda Biomedical Centre, Kigali, Rwanda

**Keywords:** Diabetes-39, D-39, Diabetes mellitus, Quality of life, Validation, Rwanda, Sub-Sahara Africa

## Abstract

**Background:**

In recent years, more importance is being given to the assessment of quality of life (QoL) among diabetic patients as a measure of their health and the goal of all health interventions. Other studies have reported a high prevalence of diabetes-related effects on; however, there is a knowledge gap in the region of Sub-Saharan Africa, as is the case for Rwanda, where the prevalence of diabetes is expected to rise over the next decade. The aim of this study is to report on the translation and cultural adaptation of the Diabetes-39 (D-39) questionnaire into the Kinyarwanda and its psychometric properties among diabetic patients in Rwanda.

**Methods:**

The D-39 questionnaire—a five-scale, disease-specific QoL questionnaire—was translated from English to Kinyarwanda, then back-translated to English. A consensus meeting discussed discrepancies and agreed on changes. Interviews were conducted with 26 participants before producing a final version. For the psychometric evaluation, the adapted version was administered to 309 patients with diabetes mellitus. Participants either came from a separate cluster-randomised controlled trial or were recruited ad hoc for this study. The evaluation included testing internal consistency, known group validity, and construct validity.

**Results:**

Participants’ mean age was 51 ± 12.7 years with a predominance of women (64%) in the sample. All five scales of the questionnaire showed a good internal consistency, with composite reliability of above 0.7. The five-factor model of the questionnaire was fitted to the 39 items. Although the fit was not exact, there was a satisfactory approximate fit (CFI = 0.93, TLI = 0.92, RMSEA = 0.05). There was a good discriminant validity except for the “social burden” and “anxiety and worry” scales (inter-factor correlation = 0.80).

**Conclusions:**

Diabetes-39 is a questionnaire developed in English that was adapted and translated into Kinyarwanda. The Kinyarwanda version of D-39 is a reliable and valid instrument to measure QoL among diabetic patients in Rwanda. The questionnaire can be helpful in research and clinical practice improving health outcomes for patients with diabetes in Rwanda and other Kinyarwanda-competent areas in the sub-region. However, certain cross-cultural differences should be considered.

**Supplementary Information:**

The online version contains supplementary material available at 10.1186/s12955-022-02034-5.

## Introduction

It is estimated by the World Health Organisation (WHO) that, in 2016, diabetes mellitus (DM) was a top seven cause of death [1]. DM is a life-changing disease with a high incidence of micro- and macrovascular complications [2]. These include neuropathy, nephropathy, retinopathy, peripheral vascular disease, coronary heart disease, and stroke. These complications are associated with high morbidity and mortality, which markedly reduce the quality of life (QoL) of the patient [3]. QoL is defined by the WHO as “an individual’s perception of their position in life in the context of the culture and value systems in which they live and in relation to their goals, expectations, standards and concerns” [4]. Assessing QoL helps in uncovering the needs of patients, in setting up preventative programmes, and in planning service delivery. Unfortunately, there is still limited evidence on ‘QoL’ of diabetes patients in Sub-Saharan Africa region as compared to the large number of studies done in higher-income countries. In Rwanda, the prevalence of DM has been estimated at 5.1% [5]. A sharp increase in the prevalence on-communicable chronic diseases is anticipated over the next decade owing to urbanization and increasingly sedentary lifestyles. Much evidence has already been generated elsewhere on the impact of diabetes on QoL as well as its associations with socio-demographic characteristics such as gender, age, education, and income; clinical factors such as severity and management of the disease; lifestyle and diet; as well as acute and chronic complications [6–10].

The increasing understanding of the importance of measuring QoL in diabetics is driving research into specific interventions and into QoL management in clinical settings [11]. The Diabetes-39 (D-39) questionnaire is a widely-used self-reporting tool, which has been significantly associated with glycaemic control, adherence to treatment and complications, and has been linked to other associated constructs of QoL [12, 13].

There are a number of existing tools—both generic and disease-specific—for measuring QoL in diabetes [14]. Generic instruments are used in the general population to measure a wide range of domains applicable to a variety of health states, conditions, and diseases. The symptoms disease-specific instruments can include the most important aspects of health, as considered by patients or clinicians [15]. Because disease-specific instruments are more focused, they can be more responsive to changes in health and provide a more detailed and accurate assessment of patients concerns. Among generic instruments for diabetes, the Medical Outcomes Study ‘Short Form (36) Health Survey’ (or SF-36) is commonly-used, but the D-39 is the preferred instrument, as it has good psychometric properties [16]. The D-39 has been translated into multiple languages, has high internal reliability and good responsiveness to change, and has been used in a wide range of interventions [11, 14–18].

To the best of our knowledge, in all of Africa, the D-39 questionnaire has only been translated and adapted into Arabic [19, 20]. There is no validated version of the D-39 questionnaire for the Sub-Saharan African context, which includes Rwanda. The aim of this paper, therefore, was to report on the translation and cultural adaption of the D-39 questionnaire into a local language- Kinyarwanda, and to evaluate its psychometric properties.

## Material and methods

### The diabetes-39 questionnaire

The instrument used was the D-39 questionnaire, a multidimensional scale developed in United States of America [21], which consists of 39 items grouped in five dimensions: Energy and mobility (15 items), diabetes control (12), social burden (5), anxiety and worry (4), and sexual functioning (3). The D-39 is used to assess the QoL of patients with type 1 and type 2 diabetes; regardless of their treatment regimen [16]. Patient themselves could rate their QoL during the last month for each item. Each item can be answered using a seven-point scale ranging from 0.5 (not affected at all) to 7.5 (extremely affected). Each of the five dimensions were summed up, and the resulting raw scores were transformed into scales ranging from 0 to 100 using a linear transformation: (raw score − minimum value)/(maximum value − minimum value) × 100 according to the developer’s instructions. The questionnaire also includes two supplementary “overall ratings”, in which respondents use the same seven-box Likert scale to evaluate their perceived overall QoL (ranging from “lowest quality” to “highest quality”) and the severity of their diabetes (ranging from “not severe at all” to “extremely severe”). The self-rating overall health status was also evaluated with a single item asking participants to rate their overall health using a five-point Likert scale [22]. Participants responded to this item from ‘’one = very poor’’; ‘’two = poor’’; ‘’three = moderate’’; ‘’four = good’’ and ‘’ five = very good).

### Translation and cross-cultural adaptation procedures

#### Translation of D-39 to Kinyarwanda, back-translation and consensus version in English

Permission to use the D-39 was obtained from the D-39 developers. Then, for the translation of the questionnaire, we used a standard approach [23] coupled with known steps in the process of adaption [24, 25]. The translation was carried out by two native Rwandans. One of the translators possess a university degree in English literature and has twelve years of work experience, while the other is a medical doctor with work experience of seven years, who also has taught English for more than eight years. Both have a certificate of proficiency in English, and they translated the questionnaire into Kinyarwanda independently, following an ‘item intent’ guide. The two translations were synthesised into one, addressing any discrepancies. The Kinyarwanda back-translation of Kinyarwanda questions into English were done by two English native speakers. One of the back-translators has a university degree in International Studies, while the other one has studied linguistics, African studies and computational linguistics. Both back-translators had excellent language skills in Kinyarwanda. They back-translated the Kinyarwanda questions into English, while blinded to the original version. Subsequently, the two backward translations were reconciled into one.

#### Assessment by expert committee

An expert committee was set up comprised of seven members, including two Rwandan forward translators, one of the back-translators, an epidemiologist, a local bilingual representative, and the two researchers conducting the study. All members of the committee were fluent in English. The aim was to appraise the results of the translations, evaluating their semantic, idiomatic, experiential and conceptual equivalence, and produce a pre-final version. A report was prepared providing an account of these steps, the controversial items, and the ways they were resolved in the consensus translation. The report and the pre-final version were shared with the questionnaire developer, and consent was received.

#### Pre-testing

The pre-final version was assessed by conducting interviews using a sample of patients (n = 26) with DM. The objective was to evaluate patient comprehension of the translated questions and the answer categories whether respondents could retrieve relevant information from memory, the effort required to answer the degree of interest and social desirability bias. To attain maximum variability of the participants, the interviews were conducted in four different hospitals. After each round, modifications were proposed for some items, based on the interview transcripts and notes. A new iteration of the questionnaire was then prepared and tested in the following round. Lastly, a final version was produced, and a report was made available to the original D-39 developers.

### Psychometric properties and statistical analysis

#### Study participants, data collection and sample size

Evaluating Psychometric Properties of D-39 was part of a cluster-randomised controlled trial (RCT), that aimed at determining the efficacy of an integrated mobile-health and community-health-worker programme for the management of diabetes in primary healthcare in Rwanda. The protocol of this RCT (ClinicalTrials.gov registration: NCT03376607) consists of a mixed-methods study, and has been published elsewhere [26].

For the purpose of conducting the confirmatory factor analysis (CFA), at least 200 participants would be necessary [24, 26–28]. The power analysis of the RCT indicated a sample size of 324 participants, which was also adequate for conducting the CFA. Nonetheless, the pre-enrolment screening revealed that a sufficient number of patients living with diabetes could not be recruited in the specific recruitment areas selected for the RCT [26]. Furthermore, logistical challenges impeded the prompt activation of the last two of the nine hospitals (Kabutare and Ruhango).

For these reasons an additional sample was also recruited for the purposes of the evaluation, following the inclusion criteria of the RCT: patients aged 21–80 years and diagnosed with DM at least six months prior to study onset. This supplementary cohort consisted of patients residing in additional zones in the catchment areas of same hospitals, except for the hospitals of Kibungo, Kibuye and Kinihira, where the number of patients was particularly low. The recruitment was carried out between June and December 2019. Exclusion criteria for both samples were illiteracy, severe hearing or visual impairments, severe mental health conditions and pregnancy, or in the post-partum period. The classification of DM type was based on the patients’ clinical records available at the hospitals. As the precise date of the diagnosis of DM was unknown for some participants, only those with at least one year of diagnosis were included so as to limit the effect of the emotional distress linked to recent diagnosis [18, 21]. *Reliability analysis and known groups validity* To assess internal reliability Cronbach’s α and composite reliability were calculated. Mean differences in total score and in the scales of the model with the closest fit were investigated across socio-demographic and clinical groups with Mann–Whitney U test. Effect sizes were calculated based on z values; r of 0.10, 0.30 and 0.50 were interpreted as small, medium and large effects respectively [29]. For continuous variables, Spearman’s correlation was used to determine which of them were associated with the total score and scales. Correlation coefficients below 0.4 were considered as weak, those between 0.4 and 0.7 as moderate, and those above 0.7 as strong [30, 31].

*Construct and discriminant validity analysis* To assess construct validity, a CFA was conducted. The five-factor model was fitted to the 39 items of the questionnaire with no cross-loadings or correlated errors terms. The estimation strategy, in line with the standard underlying assumptions of the CFA [32], is reliable, given the relatively different nature of the 5 dimensions (energy and mobility, diabetes control, social burden, anxiety and worry, and sexual functioning) used to group the 39 items under analysis.

The discriminant validity of D-39 was studied by calculating inter-factor correlations. Inter-factor correlations have been included in Table 5, supporting the assumption of a sufficient discriminant validity for all scales. The weighted least square mean and variance adjusted (WLSMV) estimator was used in the CFA. The root mean square error of approximation (RMSEA), the comparative fit index (CFI), and the Tucker Lewis index (TLI) were used to examine the approximate model fit. For RMSEA, values of less than 0.05 were indicative of a close fit and those between 0.05 and 0.08 were interpreted as adequate fit [27, 28]. The 90% confidence intervals of RMSEA were also evaluated, as they should be less than 0.05 for the lower bound and no worse than 0.08 for the upper one [27]. For CFI and TLI, values of 0.90 and above were regarded as acceptable fit [27, 28]. Hu’s and Bentler’s recommendation of raising such cut-offs to 0.95 was also taken into account [27, 33]. The relative χ2 was also calculated and a value of 2 or less was deemed adequate [27]. Finally, although the weighted root mean-square residual (WRMR) was computed and values of 1 or lower were considered a good fit, the experimental nature of this statistic thwarted drawing conclusions based on it [27, 28, 34].

Statistical analyses were performed using Stata version 16. Mplus version 7, and JASP version 16.2.

### Ethical and research clearance

The study protocol was developed, and research authorisation was sought from the Rwandan Health and Education Ministries. Ethical approval was obtained from the Rwanda National Ethics Committee and the Ethics Review Panel of the University of Luxembourg.

## Results

### Cultural adaptation

The expert panel evaluated all translations and reached a consensus (Additional file 1), particularly regarding the items without precise translation into Kinyarwanda**.** This include the following dimensions: Energy and Mobility (item question 10, 33, 35 and 36), Diabetes Control (item question 15 and 28) and Social Burden (item question 19 and 26). Three rounds of interviews were conducted thereafter, with a total of 26 diabetic participants: 22 women and 4 men, with the median age of 47 (IQR = 39–62), median years of completed education of 6 (IQR = 6–8) and the median years of diabetes 3.5 (IQR = 2–6). Comprehension of the translated items was good, and amendments were made to increase clarity and resolve any ambiguities. Table 1 summarises the consensus translation, and reasons for modification of the item questions of the D-39.

**Table 1:** Consensus translation and reasons for modification for the D-39 Items instrument

Dimension and number of item question in D-39 questionnaires (original in English)		Consensus translation				Reasons for modification
		Initial translation in Kinyarwanda	Backward translation in English	New proposed translation in Kinyarwanda	New backward proposed in English	
Energy and Mobility	Question 10. Restrictions on how far you can walk	‘’Uko uzitirwa ku ntera ushobora kugenda n'amaguru’’	How you are impeded in the distance that you are able to go with your legs	kugorwa n'intera y'urugendo ushobora kugenda n'amaguru	Experiencing misfortune on account of the distance of the journey you are able to go with your legs [walk]	“uzitirwa ku ntera” was not clear to the interviewees and was replaced with “kugorwa n’intera y’urugendo”
	Question 33. Having to organize your daily life around diabetes	kuba ukenera gutegura ubuzima bwawe bwa buri munsi ugendeye kuri diyabete	needing to plan your everyday life around diabetes	kugomba gutegura gahunda z’ubuzima bwawe bwa buri munsi ugendeye kuri diyabete	needing to prepare plans for your everyday life around diabetes	Some interviewees considered *to plan* as *to prepare*. To emphasise the meaning of plan/schedule, *“gahunda”* was added.*“kuba”* was interpreted as *“able to”*, and therefore was replaced with *“kugomba”* (“*must”*)
	Question 35. Restless sleep	gusinzira nturuhuke	restless sleeping	kuryama ukumva utaruhutse	sleeping in such a way that you feel you are not rested	Many participants failed to understand *“gusinzira nturuhuke*”: for them when one falls asleep, he/she gets rested
	Question 36. Walking more slowly than others	kugenda gahoro ugereranije n'abandi	walking more slowly than others	kugenda gahoro n'amaguru ugereranije n'abandi	walking more slowly than others	Some participants considered *“kugenda”* as *“progressing”* or *“developing”*. To help them understand *“walking”*, we added *“n’amaguru”* (literally means with “*one’s legs”* and is part of the natural expression for walking, *“kugenda n’amaguru”*)
Diabetes Control	Question 15. Losing control of your blood sugar levels	gutakaza ubushobozi bwo gucunga urugero rw'isukari mu maraso	losing the ability to manage the blood sugar level	kutabasha gucunga urugero rw'isukari mu maraso yawe	being incapable of managing the level of the sugar in your blood	“gutakaza ubushobozi” was not clear to the interviewees and was replaced with “kutabasha gucunga”; “yawe” (your) was missing in the consensus translation and was added
	Question 28. The need to eat at regular intervals	gukenera kongera kurya nyuma y'ibihe bingana	Needing to eat again after equal times	guhora kugomba gufata amafunguro mu bihe bimwe buri munsi	always needing to take meals at the same times every day	It was unclear for many interviewees what equal times means, and therefore it was rephrased
Social Burden	Question 19. The restrictions your diabetes places on your family and friends	ibyo diyabete yawe ibuza ku muryango n'inshuti bawe	what your diabetes denies to your family and friends	ibyo diyabete yawe ibuza ku nshuti zawe no ku muryango wawe	what your diabetes denies to your friends and to your family	The interviewees thought “bawe” (your) was wrong as they thought that it was referring only to “nshuti” (friends). We proposed a change to make it clear that the question refers to both family and friends. “ibyo” was replaced with “ibintu bitandukanye” to make the question clearer
	Question 26. Doing things that your family and friends don't do	gukora ibyo umuryango n'inshuti bawe badakora	doing things your family and friends don’t do	gukora ibintu bitandukanye kubera diyabete yawe, nk’ibyo inshuti zawe n’umuryango wawe badakora	doing different things because of your diabetes, such as things your friends and family do not do	Similar to question 19 there was confusion with the word *“bawe”* referring only to friends: this was changed to refer both to family and friends. Many participants did not understand to which things this question referred: “kubera diyabete yawe” was added to specify that the things that the respondent does is because of his/her diabetes

In the first round we used a layout similar to the original English instrument, in which the introductory phrase “During the past month how much was the quality of your life affected by:” was repeated once at the top of each page, and the questions beneath stated only the second part of the sentence (e.g. “your daily medication for your diabetes”). During that round, it was noticed that most of the interviewees could not understand the questions that referred to “how the quality of their life was affected during the past month”. To resolve this issue, a new layout was tested, in which every question was preceded with the introductory phrase, and the question was written in bold (e.g. “During the past month how much was the quality of your life affected by your daily medication for your diabetes”).

According to the developer’s scoring instructions, each item is scored with a 0.5 step depending on where the cross is placed by the participants (e.g., if a mark is placed on the right-side margin of the last box, that should be interpreted as 7.5). Hence, the effective possible scoring range for each item is between 0.5 and 7.5. However, during pretesting, we observed participants having difficulty marking with precision different parts of the box space. Consequently, we adopted a simplification of scoring by considering only the area of the seven boxes (i.e., each item could be scored from 1 to 7, with a step of 1), similarly to the method recommended in the Brazilian adaptation of D-39.

### Characteristics of the subjects

Table 2 shows the patients’ characteristics. Two hundred and five participants were included from the RCT, and 122 were recruited additionally for the purposes of the evaluation. A total of 18 patients were excluded as there were marked. The total sample (N = 309). were included in the analysis of the D-39. The mean total score of D-39 for the sample was 51 (SD = 12.7), the median was 52 (IQR = 42–60) and 64% were female. More than half of the participants were married and completed secondary level education. The mean and median years of completed education were 7.6 (SD = 12.7), and 6 (IQR = 5–9) respectively. Eighty-nine percent (88.7%) of the participants reported having type 2 diabetes according to their clinical record. Six (SD = 5.8) and five (IQR = 2–9) were the mean and median years since diagnosis of diabetes in the study population respectively. All the subjects were of Rwandan nationality and spoke Kinyarwanda.

**Table 2:** Sample characteristics of the study participants

*Gender*, *n (%)*	
Female	199 (64.4)
Male	110 (35.6)
Age, *mean (SD), median (IQR)*	51 (12.7), 52 (42–60)
Years of completed education, *mean (SD), median (IQR)*	7.6 (3.5), 6 (5–9)
*Highest degree obtained*, *n (%)*	
No formal education	20 (6.6)
Primary school	181 (59.3)
Secondary school	63 (20.7)
University degree	13 (4.3)
Vocational school	27 (8.9)
Postgraduate studies	1 (0.3)
*Employment status*, *n (%)*	
Unemployed	136 (44.2)
Employed	153 (49.7)
Retired	19 (6.2)
*Type of residence*, *n (%)*	
Urban	96 (31.3)
Semi	80 (26.1)
Rural	131 (42.7)
*Marital status*, *n (%)*	
Single	26 (8.4)
Married	175 (56.6)
Cohabitation	55 (17.8)
Divorced	4 (1.3)
Widow	44 (14.3)
Other	5 (1.6)
*Most usual living situation*, *n (%)*	
Lives alone	5 (1.6)
Has other people living with him/her	301 (98.4)
Number of people are living with him/her, *mean (SD), median (IQR)*	4.89 (2.3), 5 (3–6)
*Types of diabetes*, *n (%)*	
Type I	25 (8.3)
Type II	267 (88.7)
Unknown	9 (2.3)
Years since diagnosis, *mean (SD), median (IQR)*	6.3 (5.8), 5 (2–9)
Abilities, *mean (SD), median (IQR)* a	
Writing	3.3 (0.7), 3 (3–4)
Read and understand	3.2 (0.7), 3 (3–4)
Converse with other people and understand	3.5 (0.5), 4 (3–4)
Hear clearly	3.5 (0.6), 4 (3–4)
See things clearly	3.1 (0.7), 3 (3–4)
Do normal daily activities	3.1 (0.7), 3 (3–4)
Move about the community by himself/herself	3.6 (0.6), 4 (3–4)
Self-rated overall health, *mean (SD), median (IQR)* b	3.9 (0.6), 3 (3–4)

SD, standard deviation; IQR, interquartile range

^a^The abilities are evaluated in a four-point Likert scale (1 = cannot do at all; 2 = can do a little; 3 = can do; 4 = can do very well)

^b^Overall health was evaluated in five-point Likert scale (1 = very poor; 2 = poor; 3 = moderate; 4 = good; 5 = very good)

### Internal consistency

Table 3 shows that composite reliability for all scales was acceptable (> 0.7). Similarly, Cronbach’s α ranged from 0.72 for “anxiety and worry” to 0.90 for “sexual functioning”, and McDonald’s ω ranged from 0.73 for “anxiety and worry” to 0.90 for “sexual functioning”.

**Table 3:** Psychometric properties of the Kinyarwanda version of D-39

	No of items	Mean transformed scale	Median transformed scale	SD	Composite reliability	Cronbach’s alpha	McDonald’s omega
Diabetes control	12	40.8	40.3	18.0	0.83	0.81	0.81
Anxiety and worry	4	53.0	54.2	23.9	0.75	0.72	0.73
Social Burden	5	40.9	40.0	23.2	0.76	0.73	0.74
Sexual functioning	3	47.7	50.0	36.6	0.93	0.90	0.90
Energy and mobility	15	43.9	42.2	18.5	0.87	0.85	0.86

SD, standard deviation

The standardised factor loadings ranged from 0.39 to 0.67 for the “diabetes control” scale; from 0.54 to 0.75 for the “anxiety and worry” scale; from 0.53 to 0.72 for the “social burden” scale; from 0.90 to 0.91 for the “sexual functioning” scale, and from 0.38 to 0.71 for “energy and mobility” (Table 4).

**Table 4:** Mean, median and the standardised factor loading of all D-39 items

	Mean (SD), Median (IQR)	Standardized loading a	SE	R2
*Diabetes control*				
Question 1. Your daily medication for your diabetes	2.6 (1.8), 2 (1–4)	0.47	0.05	0.22
Question 4. Following your doctor's prescribed treatment plan for diabetes	2.5 (1.8), 2 (1–4)	0.45	0.06	0.20
Question 5. Food restrictions required to control your diabetes	3.6 (1.9), 3 (2–5)	0.50	0.05	0.24
Question 14. Having diabetes	4.4 (1.9), 5 (3–6)	0.67	0.04	0.44
Question 15. Losing control of your blood sugar levels	3.9 (2.0), 2 (4–6)	0.39	0.05	0.15
Question 17. Testing your blood sugar levels	2.9 (2.0), 2 (1–4)	0.45	0.05	0.20
Question 18. The time required to control your diabetes	3.2 (1.9), 3 (2–5)	0.55	0.04	0.30
Question 24. Getting your diabetes well controlled	3.3 (1.9), 3 (2–5)	0.64	0.04	0.41
Question 27. Keeping a record of your blood sugar levels	3.0 (2.0), 2 (1–5)	0.43	0.05	0.19
Question 28. The need to eat at regular intervals	3.9 (1.8), 4 (3–5)	0.56	0.04	0.31
Question 31. Having to organize you daily life around diabetes	3.7 (1.8), 4 (2–5)	0.67	0.04	0.44
Question 39. Diabetes in general	4.2 (1.8), 4 (3–6)	0.64	0.04	0.41
*Anxiety and worry*				
Question 2. Worries about money matters	4.6 (1.8), 5 (3–6)	0.54	0.05	0.29
Question 6. Concerns about your future	4.9 (1.9), 5 (4–7)	0.67	0.04	0.47
Question 8. Stress or pressure in your life	3.7 (2.1), 3 (2–6)	0.63	0.04	0.40
Question 22. Feeling depressed or low	3.5 (2.0), 3 (2–5)	0.75	0.04	0.56
*Social burden*				
Question 19. The restrictions your diabetes places on your family and friends	3.7 (2.0), 4 (2–5)	0.72	0.04	0.51
Question 20. Being embarrassed because you have diabetes	3.3 (2.1), 3 (1–5)	0.61	0.04	0.37
Question 26. Doing things that your family and friends don't do	3.5 (1.9), 3 (2–5)	0.65	0.04	0.43
Question 37. Being identified as a diabetic	2.7 (1.9), 2 (1–4)	0.53	0.05	0.28
Question 38. Having diabetes interfere with your family life	4.1 (2.1), 4 (2–6)	0.62	0.04	0.39
*Sexual functioning*				
Question 21. Diabetes interfering with your sex life	3.9 (2.5), 4 (1–6)	0.91	0.02	0.83
Question 23. Problems with sexual functioning	3.6 (2.4), 3 (1–6)	0.92	0.02	0.85
Question 30. A decreased interest in sex	4.1 (2.4), 4 (2–6)	0.90	0.02	0.82
*Energy and mobility*				
Question 3. Limited energy levels	4.2 (1.8), 4 (3–6)	0.66	0.03	0.44
Question 7. Other health problems besides diabetes	4.1 (2.0), 4 (2–6)	0.50	0.04	0.25
Question 9. Feelings of weakness	4.2 (1.8), 4 (3–6)	0.67	0.04	0.41
Question 10. Restrictions on how far you can walk	3.5 (2.1), 3 (2–5)	0.63	0.04	0.40
Question 11. Any daily exercises for your diabetes	3.0 (2.0), 3 (1–4)	0.51	0.05	0.26
Question 12. Loss or blurring of vision	3.7 (2.0), 4 (2–6)	0.38	0.05	0.14
Question 13. Not being able to do what you want	4.1 (2.0), 4 (2–6)	0.71	0.04	0.51
Question 16. Other illnesses besides diabetes	3.5 (1.97), 3 (2–5)	0.39	0.05	0.15
Question 25. Complications from your diabetes	3.6 (2.07), 4 (2–5)	0.64	0.04	0.42
Question 29. Not being able to do housework or other jobs around the house	3.6 (1.92), 3 (2–5)	0.70	0.03	0.50
Question 32. Needing to rest often	3.8 (1.87), 4 (2–5)	0.53	0.04	0.28
Question 33. Problems in climbing stairs or walking up steps	4.1 (2.01), 4 (2–6)	0.48	0.05	0.23
Question 34. Having trouble caring for yourself (dressing, bathing, or using the toilet)	2.1 (1.67), 1 (1–3)	0.52	0.06	0.27
Question 35. Restless sleep	3.7 (1.97), 4 (2–5)	0.50	0.05	0.25
Question 36. Walking more slowly than others	3.5 (1.93), 3 (2–5)	0.53	0.05	0.28

SD, standard deviation; IQR, interquartile range

^a^All standardised loadings were found significant (*p* < 0.001)

### Construct validity (confirmatory factor analysis)

Construct validity was assessed with CFA based on weighted least square mean and variance adjusted estimator. The five-factor model was fitted to the 39 items of the questionnaire and did not yield an exact fit (χ^2^ = 1228.6, df = 692, p < 0.0001, relative χ^2^ = 1.8); however, the fit indices indicated a satisfactory approximate fit (CFI = 0.93, TLI = 0.92, RMSEA = 0.05 (90% CI 0.046–0.055)).

There was sufficient discriminant validity for all scales with the exception of “social burden” and “anxiety and worry” with a reported inter-factor correlation of 0.8 (Table 5).

**Table 5:** Inter-factor correlations in the five dimensions of D-39

	Inter-factor correlations	SE	*p*
*Anxiety and worry*			
Diabetes control	0.69	0.044	0.000
*Social burden*			
Diabetes control	0.77	0.034	0.000
Anxiety and worry	0.80	0.038	0.000
*Sexual functioning*			
Diabetes control	0.23	0.058	0.000
Anxiety and worry	0.34	0.058	0.000
Social burden	0.35	0.059	0.000
*Energy and mobility*			
Diabetes control	0.73	0.033	0.000
Anxiety and worry	0.71	0.038	0.000
Social burden	0.67	0.042	0.000
Sexual functioning	0.29	0.051	0.000

SE, standard error; *P*, *P*-value

Table 6 demonstrates the relationships between socio-demographic variables, the five dimensions of the D-39, and the two additional “overall ratings” items. Overall, there were significant gender differences in the “diabetes control”, “anxiety and worry” and “energy and mobility” scales (small effect sizes), and “sexual functioning” scale (medium effect size). Small correlations were observed between years of completed education and the “anxiety and worry”, “social burden” and “energy and mobility” scales. The self-rated overall health was also weakly correlated with all D-39 scales but for the “sexual functioning”. Finally, “energy and mobility” differed significantly, albeit with a small effect size, between the two types of diabetes.

**Table 6:** Relationships between socio-demographic variables and the five dimensions of the D-39

	Diabetes control	Anxiety and worry	Social burden	Sexual functioning	Energy and mobility	Question X1	Question X2
*Gender*							
Female, *mean (SD)*	42.6 (17.9)	55.2 (23.0)	41.8 (23.3)	39.6 (34.1)	46.2 (18.4)	3.9 (1.3)	4.2 (1.4)
Male, *mean (SD)*	37.6 (17.3)	49.2 (25.0)	39.2 (23.0)	62.5 (35.0)	39.7 (18.4)	3.9 (1.3)	3.7 (1.4)
Mann–Whitney *U* test	**z = 2.267,** ***p* = 0.023**	**z = 2.133,** ***p* = 0.033**	z = 1.136, *p* = 0.257	***z* = *− 5.367,*** ***p* < *0.001***	**z = 3.113,** ***p* = 0.002**	z = − 0.279, *p* = 0.781	**z = 2.355,** ***p* = 0.018**
ES	**r = 0.129**	**r = 0.121**	r = 0.065	***r* = *− 0.305***	**r = 0.177**	r = − 0.016	**r = 0.134**
Age, *Spearman’s correlation*	rs = − 0.026, *p* = 0.646	rs = − 0.091, *p* = 0.110	rs = − 0.065, *p* = 0.258	rs = 0.107, *p* = 0.061	rs = 0.201, *p* < 0.001	rs = 0.050, *p* = 0.379	rs = 0.003, *p* = 0.953
Years of completed education, *Spearman’s correlation*	rs = − 0.073, *p* = 0.205	rs = − 0.212, *p* < 0.001	rs = − 0.184, *p* = 0.001	rs = − 0.051, *p* = 0.376	rs = − 0.177, *p* = 0.002	rs = 0.009, *p* = 0.869	rs = − 0.091, *p* = 0.111
*Highest degree obtained*							
No formal education or primary school, *mean (SD)*	40.5 (18.3)	55.6 (23.6)	42.4 (23.7)	47.8 (35.7)	45.6 (18.4)	3.9 (1.3)	3.9 (1.4)
Secondary school, university or vocational school, *mean (SD)*	41.8 (17.7)	48.0 (23.9)	37.7 (22.4)	47.3 (38.6)	40.8 (18.3)	4.0 (1.3)	3.9 (1.4)
Mann–Whitney U test	z = − 0.642, *p* = 0.522	**z = 2.545,** ***p* = 0.011**	z = 1.621, *p* = 0.105	z = 0.113, *p* = 0.910	**z = 2.158,** ***p* = 0.031**	z = − 0.968, *p* = 0.333	z = 0.694, *p* = 0.488
ES	r = − 0.037	**r = 0.146**	r = 0.093	r = 0.006	**r = 0.124**	r = − 0.055	r = 0.040
*Abilities, Spearman’s correlation*							
Writing	rs = − 0.151, *p* = 0.008	rs = − 0.201, *p* < 0.001	rs = − 0.153, *p* = 0.007	rs = − 0.033, *p* = 0.565	rs = − 0.259, *p* < 0.001	rs = 0.106, *p* = 0.063	rs = − 0.163, *p* = 0.004
Read and understand	rs = − 0.152, *p* = 0.008	rs = − 0.163, *p* = 0.004	rs = − 0.184, *p* = 0.001	rs = − 0.049, *p* = 0.393	rs = − 0.238, *p* < 0.001	rs = 0.095, *p* = 0.095	rs = − 0.205, *p* < 0.001
Converse with other people and understand	rs = − 0.157, *p* = 0.006	rs = − 0.102, *p* = 0.074	rs = − 0.078, *p* = 0.178	rs = − 0.099, *p* = 0.082	rs = − 0.107, *p* = 0.061	rs = 0.110, *p* = 0.054	rs = − 0.134, *p* = 0.019
Hear clearly	rs = − 0.108, *p* = 0.059	rs = − 0.027, *p* = 0.638	rs = − 0.042, *p* = 0.460	rs = − 0.043, *p* = 0.444	rs = − 0.126, *p* = 0.027	rs = − 0.047, *p* = 0.412	rs = − 0.088, *p* = 0.123
See things clearly	rs = − 0.184, *p* = 0.001	rs = − 0.048, *p* = 0.400	rs = − 0.066, *p* = 0.247	rs = − 0.044, *p* = 0.441	rs = − 0.183, *p* = 0.001	rs = 0.021, *p* = 0.710	rs = − 0.110, *p* = 0.054
Do normal daily activities	rs = − 0.168, *p* = 0.003	rs = − 0.124, *p* = 0.029	rs = − 0.164, *p* = 0.004	rs = − 0.090, *p* = 0.114	rs = − 0.289, *p* < 0.001	rs = 0.120, *p* = 0.036	rs = − 0.170, *p* = 0.003
Move about the community by himself/herself	rs = − 0.034, *p* = 0.560	rs = 0.009, *p* = 0.877	rs = − 0.015, *p* = 0.788	rs = 0.044, *p* = 0.441	rs = − 0.131, *p* = 0.022	rs = 0.043, *p* = 0.458	rs = − 0.005, *p* = 0.931
Self-rated overall health, *Spearman’s correlation*	rs = − 0.190, *p* = 0.001	rs = − 0.294, *p* < 0.001	rs = − 0.211, *p* < 0.001	rs = − 0.028, *p* = 0.618	rs = − 0.169, *p* = 0.003	rs = 0.172, *p* = 0.002	rs = − 0.176, *p* = 0.002
Very good or good, *mean (SD)*	37.2 (16.9)	44.5 (24.8)	35.8(23.0)	44.8 (36.2)	40.1 (17.3)	4.2 (1.2)	3.6 (1.4)
Moderate, poor, very poor, *mean (SD)*	42.7 (18.3)	57.7 (22.3)	43.5 (23.0)	49.2 (36.9)	45.9 (18.8)	3.8 (1.3)	4.11 (1.4)
Mann–Whitney U test	**z = 2.522,** ***p* = 0.012**	***z* = *4.499,*** *p* < ***0.000***	**z = 2.774,** ***p* = 0.005**	z = 0.961, *p* = 0.337	**z = 2.551,** ***p* = 0.011**	**z = − 3.053,** ***p* = 0.002**	**z = 2.949,** ***p* = 0.003**
ES	**r = 0.144**	***r* = *0.257***	**r = 0.158**	r = 0.055	**r = 0.146**	**r = − 0.174**	**r = 0.168**
*Types of diabetes*							
Type I, *mean (SD)*	41.2 (17.1)	59.7 (20.6)	41.9 (21.3)	37.3 (38.7)	35.6 (15.1)	3.7 (1.3)	3.8 (1.4)
Type II, *mean (SD)*	40.5 (18.2)	52.8 (24.4)	40.5 (23.5)	48.4 (36.5)	44.3 (18.5)	3.9 (1.3)	3.9 (1.4)
Mann–Whitney U test	z = 0.103, *p* = 0.919	z = 1.321, *p* = 0.188	z = 0.198, *p* = 0.844	z = − 1.356, *p* = 0.176	**z = − 2.258,** ***p* = 0.023**	z = − 0.744, *p* = 0.460	z = − 0.486, *p* = 0.629
ES	r = 0.006	r = 0.077	r = 0.012	r = − 0.079	**r = − 0.132**	r = − 0.044	r = − 0.028
Years since diagnosis, *Spearman’s correlation*	rs = 0.033, *p* = 0.569	rs = 0.061, *p* = 0.285	rs = 0.069, *p* = 0.232	rs = 0.035, *p* = 0.546	rs = 0.071, *p* = 0.215	rs = 0.028, *p* = 0.627	rs = 0.183, *p* = 0.001

SD, standard deviation; IQR, interquartile range; ES, effect size

Concerning the two “overall rating” items, the mean perceived quality of life was 3.9 (SD = 1.3) and the mean perceived severity of the disease was 3.9 (SD = 1.4). Question X2 (mean = 3.9, SD = 1.4, median = 4, IQR = 3–5).

## Discussion

Our research indicates that the tool we adapted to assess diabetic QoL was the first of its kind, being the only such tool to be tailored specifically with the Rwandan and sub-Sahara Africa cultural contexts in mind. We analysed, made cultural adaptations to, and translated the D-39 Questionnaire into Kinyarwanda. With approximately 20 million speakers, Kinyarwanda is one of the most widely-spoken bantu languages, known to have both grammatical and lexical reduplications and is a national language in Rwanda [35].

There are a number of dialects and word substitutions throughout Rwanda, and so we aimed to account for these so that the Kinyarwanda version could be understood by the majority. Sometimes, different words may be used to express a single concept, and there is precedent for this approach [35]. We aimed to assemble a varied consensus panel, in order to enable a comprehensive assessment of the translated version. The feedback from patients regarding comprehension was particularly useful in achieving consensus on highlighted discrepancies; agreement was reached not only on the wording and formulation of items, but also on the changes which needed to be made. These adaptations were intended to improve respondent understanding, and to increase consistency in responses.

The full scale showed a good internal reliability in line with previous studies [14, 15, 36, 37]. Overall, some items did not load highly in some scales (e.g., diabetes control), while others performed better (e.g., sexual functioning). Discriminant validity was assessed through inter-factor correlations. In this study, there was good discriminant validity for all scales with the exception of the “anxiety and worry” and “social burden” scale (0.80). A similar lower correlation coefficient were observed in the Brazilian study [38] for the domain “anxiety and worry” (0.21) and for the domain “social burden” (0.34) in a study from Jordan [19].

All of the D-39 domains are higher than the composite reliability standard of 0.7 that previously justified as a value to support claims of internal reliability of the instrument [37, 39]. Previous studies have shown that a Cronbach’s alpha coefficient of below 0.70 can undermine the instrument’s internal consistency [40]. For each of the five scales in the 39-item instrument, the Cronbach's coefficient alpha was calculated. The results of the D-39 item and scales tests assumption in this study showed that the internal consistency reliability Cronbach’s alpha in the diabetic population in Rwanda ranged between 0.72 and 0.92. This is similar (or higher) to those obtained in a study population of Jordan [19] (0.80 to 0.92), of the United states [21] (0.82 to 0.93 and 0.81 to 0.93 for Iowa and Carolina studies respectively) and of the Nordic countries [41] (0.83 to 0.92, 0.83 to 0.91 and 0.82 to 0.92 for Finnish, Norwegian and Danish studies respectively). The Cronbach’s alpha of this study differed from a Moroccan study [20] (0.65–0.93), and a Brazilian study [38] (0.58 to 0.85). It is worth mentioning that our sample size of 309 was approximate to the one used in the Jordan study [19] (N = 368) and higher than the studies in Brazil N = 52 and Morocco N = 92 [20, 38].

Despite the fact that a lot of effort was engaged in reaching out the communities to recruit a large sample, there have been significant logistical and systemic barriers, and this was marked as the study limitation. The presentation of diabetes specific QoL may differ between patients depending on the form of the disease, and this should be noted as a possible limitation of this study. For example, Insulin dependent diabetes mellitus patients may present with a higher fear of hypoglycaemia [42]. Although we observed no significant differences between the two forms of the disease, our sample consisted predominantly of patients with type 2 DM. Notwithstanding this, such distinctions between forms of the disease need to be treated cautiously due to the possibility of misclassification and/or atypical disease forms [42–45]. The treatment type may also cause a separate effect, particularly pertaining to the use of insulin [46]; however, information on insulin use was not collected for this study.

As it was not possible to identify another established and previously validated tool in the Rwandan population—either generic or diabetes-specific—there was a lack of testing for convergent validity, and this may also be considered a limitation of our study. Test–retest reliability was not carried out, and further research is therefore indicated. We were also unable to evaluate the correlation of D-39 with glycated haemoglobin, as in Rwanda this was not routinely measured during the time we conducted this study, and ad hoc measurements for the entire study sample were not possible. Finally, as reliable diagnoses were not easily obtained from patients’ medical records, it was difficult to effectively investigate comorbidity.

The results show the perceptions of patients and their health care providers on gaps in the readiness of the society, patients, and the health care system to ensure improved health related QoL of diabetes patients. A programme to ensure QoL would tackle many challenges that are currently being faced by diabetic patients in Rwanda while at the same time addressing the increasing prevalence of the disease in the country. Such a study would help generate new insight around factors influencing the health related QoL within the Rwandan social, cultural and demographic context [47, 48], thus informing researchers and clinical practice for better health outcomes.

## Conclusion

Diabetes-39 is a questionnaire originally developed in English which was adapted and translated into Kinyarwanda for the purposes of this study. Our results confirm that this Kinyarwanda version is a both reliable and valid instrument to measure the health related QoL of diabetic patients, and could help both researchers and clinicians in their practice to improve health outcomes for patient with diabetes in Rwanda and its sub-region. It can provide insights into the factors that impact QoL, in the context of Rwandan values and culture, and also for the purposes of assessment in disease management. Further scale assessment, using larger samples with a more diverse population across sub-Saharan Africa, would strengthen the evidence for the viability of this questionnaire as a health related QoL tool for diabetic patients.

## Supplementary Information

**Additional file 1: Appendix**. The Kinyarwanda Version of the Diabetes-39 (D-39) Questionnaire.

## Data Availability

The data used to support the findings of this study are restricted by the Government of Rwanda, and cannot be released or shared partially or totally with third parties without the written permission of the Rwanda Biomedical Centre. Data are available from the corresponding author for researchers who meet the criteria for access to confidential data, and only after authorisation from the Rwanda Biomedical Centre.
