# Supplementary material for: Cultural adaptation and psychometric evaluation of the Kinyarwanda version of the diabetes-39 (D-39) questionnaire
Source: Health Qual Life Outcomes. 2022 Aug 16;20:122. doi: 10.1186/s12955-022-02034-5 (PMC9382791; doi:10.1186/s12955-022-02034-5)
Supplement: Supplementary file 1 — Additional file 1: Appendix. The Kinyarwanda Version of the Diabetes-39 (D-39) Questionnaire. [file 12955_2022_2034_MOESM1_ESM.docx]

Additional file 1: Appendix

The Kinyarwanda Version of the Diabetes-39 (D-39) Questionnaire

IMIBEREHO KURI DIYABETE

Imibereho y'umuntu ikorwaho n'ibintu byinshi. Muri ibyo harimo ubuzima, amahirwe yo kwidagadura no kuruhuka, inshuti n'umuryango, akazi ndetse imihangayiko n'ingorane bya diyabete. Uru rutonde rw'ibibazo rwakozwe kugirango rudufashe kumenya cyo diyabete itera ku mibereho y'ubuzima bw'abayirwaye.

Uko ibi bibazo bisubizwa:

- Kuri buri kibazo mu bikurikira, turashaka kumenya uburyo imibereho yawe yagezweho n'ingaruka. Subiza ibibazo ushyira akamenyetso (X) ahantu hamwe ku murongo ukurikira buri kibazo. Umurongo uhera ku mubare 1, akamenyetso washyira aho kaba kavuga ko imibereho yawe itagezweho n'ingaruka na gato. Umurongo urangirira kuri 7 akamenyetso aho kerekana ko imibereho yagezweho n'ingaruka bikabije. Shyira aka kamenyetso aho wumva kerekana neza uko imibereho yawe yagezweho n'ingaruka mu kwezi gushize.
- Ni ngombwa cyane ko usubiza buri kibazo.Ariko bimwe mu bibazo bibaza ku buzima bwawe bwite bishobora kuba wowe ntacyo bitakwerekeyeho. Ibitakwerekeyeho bireke ujye kubindi bibazo bikurikiyeho. Ibizubizo byose bizafatwa nk'ibanga.

**Urugero**

Niba utekereza ko "ihumana ry'ikirere" ryagize ingaruka ku mibereho yawe ku rugero runaka ariko bidakabije cyane, ushobora gushyira akamenyetso ku murongo nk'uko aha bigaragara.

1. Mu kwezi gushize ni gute imibereho yawe yaba yaragizweho ingaruka na **ihumana ry'ikirere**

| Nta ngaruka na gato | 1 | 2 | 3 | 4 | 5 | 6 | 7 | Ingaruka zambayeho nyinshi bikomeye |
| --- | --- | --- | --- | --- | --- | --- | --- | --- |

Niba ugiye gusubiza ikibazo kimeze kimwe, akamenyetso wagashyira he? Niba utekereza ko waba waragezweho n'ingaruka zo guhumana k'umwuka kurusha umuntu uri mu rugero rwatanzwe, urashyira akamenyetso kawe ahantu iburyo bw'akamenyetso kariho. Ariko niba utekereza ko wagezweho n'ingaruka biri munsi, akamenyetso kawe uragashyira ahantu ibumoso bw'akamenyetso kariho. Mu kwitoza, ngaho shyira akamenyetso kawe ku murongo.

**Subiza ibibazo bikurikira. Niba ufite ikibazo ku buryo usubiza ibi bibazo, wabaza umuforomo wawe cyangwa muganga.**

1. Mu kwezi gushize ni gute imibereho yawe yaba yaragizweho ingaruka z’**imiti yawe ya buri munsi ya diyabete**

| Nta ngaruka na gato | 1 | 2 | 3 | 4 | 5 | 6 | 7 | Ingaruka zambayeho nyinshi bikomeye |
| --- | --- | --- | --- | --- | --- | --- | --- | --- |

1. Mu kwezi gushize ni gute imibereho yawe yaba yaragizweho ingaruka z’**impungenge zigendanye n'amafaranga**

| Nta ngaruka na gato | 1 | 2 | 3 | 4 | 5 | 6 | 7 | Ingaruka zambayeho nyinshi bikomeye |
| --- | --- | --- | --- | --- | --- | --- | --- | --- |

1. Mu kwezi gushize ni gute imibereho yawe yaba yaragizweho ingaruka zo **kugira intege nke**

| Nta ngaruka na gato | 1 | 2 | 3 | 4 | 5 | 6 | 7 | Ingaruka zambayeho nyinshi bikomeye |
| --- | --- | --- | --- | --- | --- | --- | --- | --- |

1. Mu kwezi gushize ni gute imibereho yawe yaba yaragizweho ingaruka zo **gukurikiza gahunda z'ubuvuzi bwa diyabete zagenwe na muganga**

| Nta ngaruka na gato | 1 | 2 | 3 | 4 | 5 | 6 | 7 | Ingaruka zambayeho nyinshi bikomeye |
| --- | --- | --- | --- | --- | --- | --- | --- | --- |

1. Mu kwezi gushize ni gute imibereho yawe yaba yaragizweho ingaruka z’**ibyo wabujijwe kurya kugirango diyabete yawe igume ku murongo**

| Nta ngaruka na gato | 1 | 2 | 3 | 4 | 5 | 6 | 7 | Ingaruka zambayeho nyinshi bikomeye |
| --- | --- | --- | --- | --- | --- | --- | --- | --- |

1. Mu kwezi gushize ni gute imibereho yawe yaba yaragizweho ingaruka zo **guhangayikishwa n'ejo hazaza hawe**

| Nta ngaruka na gato | 1 | 2 | 3 | 4 | 5 | 6 | 7 | Ingaruka zambayeho nyinshi bikomeye |
| --- | --- | --- | --- | --- | --- | --- | --- | --- |

1. Mu kwezi gushize ni gute imibereho yawe yaba yaragizweho ingaruka z’**ibindi bibazo bifata ku buzima bitari diyabete**

| Nta ngaruka na gato | 1 | 2 | 3 | 4 | 5 | 6 | 7 | Ingaruka zambayeho nyinshi bikomeye |
| --- | --- | --- | --- | --- | --- | --- | --- | --- |

1. Mu kwezi gushize ni gute imibereho yawe yaba yaragizweho ingaruka z’**inkeke cyangwa umutwaro mu mibereho yawe**

| Nta ngaruka na gato | 1 | 2 | 3 | 4 | 5 | 6 | 7 | Ingaruka zambayeho nyinshi bikomeye |
| --- | --- | --- | --- | --- | --- | --- | --- | --- |

1. Mu kwezi gushize ni gute imibereho yawe yaba yaragizweho ingaruka zo **kwiyumvamo intege nke**

| Nta ngaruka na gato | 1 | 2 | 3 | 4 | 5 | 6 | 7 | Ingaruka zambayeho nyinshi bikomeye |
| --- | --- | --- | --- | --- | --- | --- | --- | --- |

1. Mu kwezi gushize ni gute imibereho yawe yaba yaragizweho ingaruka zo **kugorwa n'intera y'urugendo ushobora kugenda** **n'amaguru**

| Nta ngaruka na gato | 1 | 2 | 3 | 4 | 5 | 6 | 7 | Ingaruka zambayeho nyinshi bikomeye |
| --- | --- | --- | --- | --- | --- | --- | --- | --- |

1. Mu kwezi gushize ni gute imibereho yawe yaba yaragizweho ingaruka z’**imyitozo ngororamubiri iyo ariyo yose ya buri munsi kubera diyabete**

| Nta ngaruka na gato | 1 | 2 | 3 | 4 | 5 | 6 | 7 | Ingaruka zambayeho nyinshi bikomeye |
| --- | --- | --- | --- | --- | --- | --- | --- | --- |

1. Mu kwezi gushize ni gute imibereho yawe yaba yaragizweho ingaruka zo **guhuma cyangwa kutabona neza**

| Nta ngaruka na gato | 1 | 2 | 3 | 4 | 5 | 6 | 7 | Ingaruka zambayeho nyinshi bikomeye |
| --- | --- | --- | --- | --- | --- | --- | --- | --- |

1. Mu kwezi gushize ni gute imibereho yawe yaba yaragizweho ingaruka zo **kutabasha gukora icyo ushaka**

| Nta ngaruka na gato | 1 | 2 | 3 | 4 | 5 | 6 | 7 | Ingaruka zambayeho nyinshi bikomeye |
| --- | --- | --- | --- | --- | --- | --- | --- | --- |

1. Mu kwezi gushize ni gute imibereho yawe yaba yaragizweho ingaruka zo **kurwara diyabete**

| Nta ngaruka na gato | 1 | 2 | 3 | 4 | 5 | 6 | 7 | Ingaruka zambayeho nyinshi bikomeye |
| --- | --- | --- | --- | --- | --- | --- | --- | --- |

1. Mu kwezi gushize ni gute imibereho yawe yaba yaragizweho ingaruka zo **kutabasha gucunga urugero rw'isukari mu maraso yawe**

| Nta ngaruka na gato | 1 | 2 | 3 | 4 | 5 | 6 | 7 | Ingaruka zambayeho nyinshi bikomeye |
| --- | --- | --- | --- | --- | --- | --- | --- | --- |

1. Mu kwezi gushize ni gute imibereho yawe yaba yaragizweho ingaruka z’**ubundi burwayi butari diyabete**

| Nta ngaruka na gato | 1 | 2 | 3 | 4 | 5 | 6 | 7 | Ingaruka zambayeho nyinshi bikomeye |
| --- | --- | --- | --- | --- | --- | --- | --- | --- |

1. Mu kwezi gushize ni gute imibereho yawe yaba yaragizweho ingaruka zo **gupimisha urugero rw'isukari iri mu maraso yawe**

| Nta ngaruka na gato | 1 | 2 | 3 | 4 | 5 | 6 | 7 | Ingaruka zambayeho nyinshi bikomeye |
| --- | --- | --- | --- | --- | --- | --- | --- | --- |

1. Mu kwezi gushize ni gute imibereho yawe yaba yaragizweho ingaruka z’**igihe gikenewe mu gucunga diyabete yawe**

| Nta ngaruka na gato | 1 | 2 | 3 | 4 | 5 | 6 | 7 | Ingaruka zambayeho nyinshi bikomeye |
| --- | --- | --- | --- | --- | --- | --- | --- | --- |

1. Mu kwezi gushize ni gute imibereho yawe yaba yaragizweho ingaruka z’**ibyo diyabete yawe ibuza ku nshuti zawe no ku muryango wawe**

| Nta ngaruka na gato | 1 | 2 | 3 | 4 | 5 | 6 | 7 | Ingaruka zambayeho nyinshi bikomeye |
| --- | --- | --- | --- | --- | --- | --- | --- | --- |

1. Mu kwezi gushize ni gute imibereho yawe yaba yaragizweho ingaruka zo **guterwa ipfunwe no kurwara diyabete**

| Nta ngaruka na gato | 1 | 2 | 3 | 4 | 5 | 6 | 7 | Ingaruka zambayeho nyinshi bikomeye |
| --- | --- | --- | --- | --- | --- | --- | --- | --- |

1. Mu kwezi gushize ni gute imibereho yawe yaba yaragizweho ingaruka za **diyabete ikubangamira mu buzima bw'imibonano mpuzabitsina**

| Nta ngaruka na gato | 1 | 2 | 3 | 4 | 5 | 6 | 7 | Ingaruka zambayeho nyinshi bikomeye |
| --- | --- | --- | --- | --- | --- | --- | --- | --- |

1. Mu kwezi gushize ni gute imibereho yawe yaba yaragizweho ingaruka zo **kwiyumvamo ukwiheba cyangwa se gucika intege**

| Nta ngaruka na gato | 1 | 2 | 3 | 4 | 5 | 6 | 7 | Ingaruka zambayeho nyinshi bikomeye |
| --- | --- | --- | --- | --- | --- | --- | --- | --- |

1. Mu kwezi gushize ni gute imibereho yawe yaba yaragizweho ingaruka z’**ibibazo by'imikorere y'igitsina**

| Nta ngaruka na gato | 1 | 2 | 3 | 4 | 5 | 6 | 7 | Ingaruka zambayeho nyinshi bikomeye |
| --- | --- | --- | --- | --- | --- | --- | --- | --- |

1. Mu kwezi gushize ni gute imibereho yawe yaba yaragizweho ingaruka zo **kubasha gucunga neza diyabete yawe**

| Nta ngaruka na gato | 1 | 2 | 3 | 4 | 5 | 6 | 7 | Ingaruka zambayeho nyinshi bikomeye |
| --- | --- | --- | --- | --- | --- | --- | --- | --- |

1. Mu kwezi gushize ni gute imibereho yawe yaba yaragizweho ingaruka z’**ibindi bibazo by'uburwayi biturutse kuri diyabete yawe**

| Nta ngaruka na gato | 1 | 2 | 3 | 4 | 5 | 6 | 7 | Ingaruka zambayeho nyinshi bikomeye |
| --- | --- | --- | --- | --- | --- | --- | --- | --- |

1. Mu kwezi gushize ni gute imibereho yawe yaba yaragizweho ingaruka zo **gukora ibintu bitandukanye kubera diyabete yawe, nk’ibyo inshuti zawe n’umuryango wawe badakora**

| Nta ngaruka na gato | 1 | 2 | 3 | 4 | 5 | 6 | 7 | Ingaruka zambayeho nyinshi bikomeye |
| --- | --- | --- | --- | --- | --- | --- | --- | --- |

1. Mu kwezi gushize ni gute imibereho yawe yaba yaragizweho ingaruka zo **kubika amakuru y'ibipimo by'isukari iri mu maraso yawe**

| Nta ngaruka na gato | 1 | 2 | 3 | 4 | 5 | 6 | 7 | Ingaruka zambayeho nyinshi bikomeye |
| --- | --- | --- | --- | --- | --- | --- | --- | --- |

1. Mu kwezi gushize ni gute imibereho yawe yaba yaragizweho ingaruka zo **guhora kugomba gufata amafunguro mu bihe bimwe buri munsi**

| Nta ngaruka na gato | 1 | 2 | 3 | 4 | 5 | 6 | 7 | Ingaruka zambayeho nyinshi bikomeye |
| --- | --- | --- | --- | --- | --- | --- | --- | --- |

1. Mu kwezi gushize ni gute imibereho yawe yaba yaragizweho ingaruka zo **kudashobora gukora imirimo yo mu rugo cyangwa indi mirimo ikorerwa hafi mu rugo**

| Nta ngaruka na gato | 1 | 2 | 3 | 4 | 5 | 6 | 7 | Ingaruka zambayeho nyinshi bikomeye |
| --- | --- | --- | --- | --- | --- | --- | --- | --- |

1. Mu kwezi gushize ni gute imibereho yawe yaba yaragizweho ingaruka zo **kugabanuka k'ubushake bw'imibonano mpuzabitsina**

| Nta ngaruka na gato | 1 | 2 | 3 | 4 | 5 | 6 | 7 | Ingaruka zambayeho nyinshi bikomeye |
| --- | --- | --- | --- | --- | --- | --- | --- | --- |

1. Mu kwezi gushize ni gute imibereho yawe yaba yaragizweho ingaruka zo **kugomba gutegura gahunda z’ubuzima bwawe bwa buri munsi ugendeye kuri diyabete**

| Nta ngaruka na gato | 1 | 2 | 3 | 4 | 5 | 6 | 7 | Ingaruka zambayeho nyinshi bikomeye |
| --- | --- | --- | --- | --- | --- | --- | --- | --- |

1. Mu kwezi gushize ni gute imibereho yawe yaba yaragizweho ingaruka zo **gukenera kuruhuka kenshi**

| Nta ngaruka na gato | 1 | 2 | 3 | 4 | 5 | 6 | 7 | Ingaruka zambayeho nyinshi bikomeye |
| --- | --- | --- | --- | --- | --- | --- | --- | --- |

1. Mu kwezi gushize ni gute imibereho yawe yaba yaragizweho ingaruka z’**ibibazo mu guterera ahazamuka**

| Nta ngaruka na gato | 1 | 2 | 3 | 4 | 5 | 6 | 7 | Ingaruka zambayeho nyinshi bikomeye |
| --- | --- | --- | --- | --- | --- | --- | --- | --- |

1. Mu kwezi gushize ni gute imibereho yawe yaba yaragizweho ingaruka zo **kugorwa no kwiyitaho (kwiyambika, koga, cyangwa kujya ku musarane)**

| Nta ngaruka na gato | 1 | 2 | 3 | 4 | 5 | 6 | 7 | Ingaruka zambayeho nyinshi bikomeye |
| --- | --- | --- | --- | --- | --- | --- | --- | --- |

1. Mu kwezi gushize ni gute imibereho yawe yaba yaragizweho ingaruka zo **kuryama ukumva utaruhutse**

| Nta ngaruka na gato | 1 | 2 | 3 | 4 | 5 | 6 | 7 | Ingaruka zambayeho nyinshi bikomeye |
| --- | --- | --- | --- | --- | --- | --- | --- | --- |

1. Mu kwezi gushize ni gute imibereho yawe yaba yaragizweho ingaruka zo **kugenda gahoro n'amaguru ugereranije n'abandi**

| Nta ngaruka na gato | 1 | 2 | 3 | 4 | 5 | 6 | 7 | Ingaruka zambayeho nyinshi bikomeye |
| --- | --- | --- | --- | --- | --- | --- | --- | --- |

1. Mu kwezi gushize ni gute imibereho yawe yaba yaragizweho ingaruka zo **kumenyekana nk'umurwayi wa diyabete**

| Nta ngaruka na gato | 1 | 2 | 3 | 4 | 5 | 6 | 7 | Ingaruka zambayeho nyinshi bikomeye |
| --- | --- | --- | --- | --- | --- | --- | --- | --- |

1. Mu kwezi gushize ni gute imibereho yawe yaba yaragizweho ingaruka za **diyabete yawe ibangamira imibereho yanyu nk'umuryango**

| Nta ngaruka na gato | 1 | 2 | 3 | 4 | 5 | 6 | 7 | Ingaruka zambayeho nyinshi bikomeye |
| --- | --- | --- | --- | --- | --- | --- | --- | --- |

1. Mu kwezi gushize ni gute imibereho yawe yaba yaragizweho ingaruka za **diyabete muri rusange**

| Nta ngaruka na gato | 1 | 2 | 3 | 4 | 5 | 6 | 7 | Ingaruka zambayeho nyinshi bikomeye |
| --- | --- | --- | --- | --- | --- | --- | --- | --- |

**ISUZUMARWEGO RUSANGE**

1. Ushyireho akamenyetso "X" kuri uyu murongo uri munsi werekana **ikigereranyo k'imibereho yawe muri rusange**

| Imibereho mibi cyane | 1 | 2 | 3 | 4 | 5 | 6 | 7 | Imibereho myiza cyane |
| --- | --- | --- | --- | --- | --- | --- | --- | --- |

1. Ushyireho akamenyetso ka "X" ku murongo ukurikira werekane **uko wumva uburembe bwawe na diyabete yawe bumeze**

| Nta burembe na gato | 1 | 2 | 3 | 4 | 5 | 6 | 7 | Uburembe bukabije cyane |
| --- | --- | --- | --- | --- | --- | --- | --- | --- |
